# Prevalence of problematic feeding in young children born prematurely: a meta-analysis

**DOI:** 10.1186/s12887-021-02574-7

**Published:** 2021-03-06

**Authors:** Britt Frisk Pados, Rebecca R. Hill, Joy T. Yamasaki, Jonathan S. Litt, Christopher S. Lee

**Affiliations:** 1 Boston College William F. Connell School of Nursing, 140 Commonwealth Avenue, Maloney Hall 268, Chestnut Hill, MA 02467 USA; 2 CHA Hollywood Presbyterian Medical Center, Los Angeles, CA USA; 3 Beth Israel Deaconess Medical Center, Boston Children’s Hospital, Harvard Medical School, Harvard TH Chan School of Public Health, Boston, MA USA

**Keywords:** Infant, premature, Child, Bottle feeding, Breast feeding, Feeding and eating disorders, Feeding behavior

## Abstract

**Background:**

Difficulties related to eating are often reported in children born preterm. The objective of this study was to quantitatively synthesize available data on the prevalence of problematic feeding in children under 4 years of age who were born preterm.

**Methods:**

Literature was identified from PubMed, CINAHL, and PsycInfo. The search was limited to English language and publication years 2000–2020. To be included in the meta-analysis, the article had to report the prevalence of problematic oral feeding within a population of children born prematurely (< 37 weeks’ gestation), and the child age at the time of study had to be between full-term corrected age and 48 months. For studies meeting inclusion criteria, the following data were extracted: sample size and subsamples by gestational age and/or child age at time of study; definition of problematic feeding; measures used for assessment of feeding; gestational age at time of birth of sample; child age at time of study; exclusion criteria for the study; and prevalence of problematic feeding. Random-effects meta-analyses were performed to estimate the prevalence of problematic feeding across all studies, by gestational age at birth, and by child age at time of study.

**Results:**

There were 22 studies that met inclusion criteria. Overall prevalence of problematic feeding (*N* = 4381) was 42% (95% CI 33–51%). Prevalence was neither significantly different across categories of gestational age nor by child age at the time of study. Few studies used psychometrically-sound assessments of feeding.

**Conclusion:**

Problematic feeding is highly prevalent in prematurely-born children in the first 4 years of life regardless of degree of prematurity. Healthcare providers of children born preterm should consider screening for problematic feeding throughout early childhood as a potential complication of preterm birth.

**Systematic review registration number:**

Not applicable.

**Supplementary Information:**

The online version contains supplementary material available at 10.1186/s12887-021-02574-7.

## Background

Feeding difficulties are a common complication experienced by preterm infants hospitalized in the neonatal intensive care unit [1]. Discharge from the hospital is often dependent on infants achieving sufficient oral feeding skills to accomplish appropriate growth, and feeding difficulties are a frequent reason for prolonged length of stay [1]. After discharge and through the first several years of life, infants and young children born preterm have been found to have more difficulties with feeding compared with their term-born peers [2–6].

Problematic feeding after neonatal discharge entails the child being unable or unwilling to safely eat and/or drink enough to obtain appropriate nutrition and hydration, despite the availability of food [7]. Specific symptoms of problematic feeding change over the first several years of life as children transition from a liquid-based diet (i.e., human milk or infant formula) [8, 9] to early complementary foods, and then to more complex foods [10]. As the skills required to successfully eat change, the symptoms of problems also change [7, 11]. Symptoms of problematic feeding may include behaviors such as refusing to eat appropriate volumes or developmentally-appropriate varieties of foods; symptoms of dysphagia or aspiration, such as coughing, choking, gagging, or respiratory compromise; problematic feeding behaviors, such as increased stress, crying, irritability or strict requirements for mealtime success; or delayed eating skills, such as difficulty chewing [8–11].

The prevalence of problematic feeding over the first several years of life in the population of children born preterm is not well understood. Understanding the prevalence of this problem and who is at greatest risk may help healthcare providers assess risk in preterm-born children and facilitate earlier interventions. Additionally, understanding the prevalence of this problem may guide the need for additional research to improve the care of these vulnerable children.

## Methods

The primary purpose of this study was to quantitatively synthesize the prevalence of problematic feeding in children under 4 years of age who were born prematurely (< 37 weeks’ gestation). We hypothesized that problematic feeding would be more prevalent among infants born at earlier gestational ages compared to later gestational ages. We also hypothesized that prevalence of problematic feeding would be higher at younger ages and decrease in older children. The secondary aim of this study was to assess the risk of bias in measurement of problematic feeding within included studies by evaluating the psychometric properties of the feeding assessments used.

### Data sources and study eligibility

PubMed, CINAHL, and PsycInfo were searched for literature reporting on the prevalence of problematic feeding in preterm-born infants (defined as < 37 weeks gestational age at birth) with the child age at the time of study being between full-term corrected age and 48 months old. Literature identification was conducted through an iterative process of multiple database searches and reference list reviews. Databases were searched for terms including: feeding or eating, difficult* or problem or dysfunction or disorder, and premature or preterm. Limitations were placed on the search including English language, humans, published after January 1, 2000, and full text. The literature search was conducted in May 2020.

Literature was limited to that published since 2000 because significant progress has been made in the medical treatment and neuroprotection of premature infants in the last 20 years, particularly with regards to management of chronic lung disease. Because feeding is highly tied to both respiratory status and neurodevelopment, we chose to only include studies reporting on the more recent era of neonatal care.

To be included in the meta-analysis, the article had to be written in English, have the full-text available through a comprehensive global inter-library loan network, report the prevalence of problematic oral feeding within a population of children who were born prematurely (defined as < 37 weeks’ gestation), and the child age at the time of study had to be between full-term corrected age and 48 months. Articles were excluded if they reported on samples collected from a feeding clinic, in which case the sample would be biased towards problematic feeding. Studies that compared feeding in infants born prematurely to those born full-term, but did not report a prevalence of problematic feeding within the premature sample, were also excluded. Until recently, there has been no accepted definition of problematic feeding [12]. For the purposes of this study, problematic feeding was broadly defined as any type of problematic oral feeding, such as dysphagia, aspiration, problematic feeding behaviors, feeding refusal, or delayed eating skills.

### Data extraction

The following data were extracted from studies that met inclusion criteria: study author(s), year, and country of publication; sample size and subsamples by gestational age and/or child age at time of study; definition of problematic feeding; measures used for assessment of feeding; gestational age at time of birth of sample; child age at time of study; exclusion criteria for the study; and prevalence of problematic feeding. If problematic feeding was defined in more than one way and/or more than one prevalence was reported, the highest prevalence was used for the analysis. Data extraction was performed by the first author and validated by a second member of the research team (JY).

### Statistical analysis

A random-effects meta-analysis of proportions approach was used to quantify the prevalence of problematic feeding in prematurely-born children in three ways. First, an overall prevalence was calculated across all studies. In several studies, the authors reported the prevalence for more than one subsample of infants, in which case each reported prevalence was entered into the analysis separately. The highest reported prevalence of problematic feeding in the first 4 years of life for each sample was entered into the analysis. Second, prevalence was calculated and compared across studies by gestational age at birth and by child age at the time of study. To evaluate the prevalence of problematic feeding by gestational age at birth, the studies were categorized into three categories based on the gestational ages at birth of the children included in the sample. The three categories were: extremely preterm (gestational age < 28 weeks), very preterm (gestational age 28–32 weeks), and moderate to late preterm (gestational age 33–37 weeks). Studies were categorized by the mean gestational age of the sample and the highest prevalence of problematic feeding reported in the study was used. For studies that did not report a mean gestational age and only reported range, they were placed in the category of the middle of the reported range. Data from studies that reported prevalence of a widely mixed gestational age sample were excluded from the analysis of problematic feeding by gestational age at birth.

Finally, to evaluate the prevalence of problematic feeding by child age at the time of study, the prevalence of problematic feeding was estimated and compared across studies based on four age categories: full-term – 5 months corrected gestational age, 6–11 months, 12–23 months, and 24–48 months. In longitudinal studies that reported prevalence of the same sample at multiple time points, all time points were used and the highest prevalence within each age category was entered into the analysis.

For all analyses, the random-effects model was chosen to incorporate both within- and between-study heterogeneity, which was appropriate given the lack of clear definition and poor measurement of problematic feeding. Weighted estimates, taking into account precision as a function of sample size, 95% confidence intervals (CI), z-tests (i.e., summary estimate divided by standard error of the summary estimate), and associated *p*-values were calculated. Additionally, dispersion in effect size across studies (*Q*) along with an associated *p*-value, and variation in observed estimates attributable to heterogeneity (*I*^*2*^) were calculated. Comparison of meta-analytic estimates of prevalence across categories of gestational age at birth and age at assessment was made using tests of heterogeneity among subgroups. An alpha of .05 was considered statistically significant for all tests; Stata v16 (College Station, TX) was used to perform all analyses.

### Assessment of risk of bias

To assess the risk of bias in measurement of problematic feeding within included studies, the quality of feeding assessments used were evaluated by their psychometric properties. A review of the literature was conducted in PubMed, CINAHL, and PyscInfo using the name of the feeding assessment measure. Data regarding the psychometric properties of the measure were extracted (RH) and verified by a second member of the team (BP). To assess the risk of bias in sampling, the exclusion criteria for each study was evaluated. Bias was assessed qualitatively.

## Results

### Included studies

There were 22 studies that met inclusion criteria [2–5, 13–30]. Additional file 1: Figure 1 presents a Preferred Reporting Items for Systematic Reviews and Meta-Analyses (PRISMA) [31] diagram of the results of study identification, screening, inclusion, and exclusion (with reasons). Table 1 presents data extracted from included studies.

**Table 1:** Characteristics of Studies Included in Meta-Analysis

First Author, Year of Publication, Country	Preterm Sample Size	Mean GA of Sample in weeks ± SD or (range)	Child Age at Time of Study	Exclusion Criteria	Feeding Assessment	Prevalence of Problematic Feeding
Adams-Chapman, 2013 [14], US	1477	26 ± 2	18–22 mos CGA	Congenital infection or anomalies	Informal/Clinical Assessment	13%
Adams-Chapman, 2015 [13], US	467	26.2 ± 1.8	18 and 30 mos CGA	Congenital infection, major malformation, or congenital syndrome	Informal/Clinical Assessment	18 mos: 47% 30 mos: 25%
Bilgin, 2016 [15], UK	73	29.4 (25–33)	Term, 3, 6, and 18 mos CGA	None described	Informal/Clinical Assessment and Faddy Eating/Food Refusal Scale	Term: 50.7% 3 mos: 20.5% 6 mos: 26% 18 mos: 57.5%
Buswell, 2009 [16], UK	15	32 5/7 (24 4/7–36 6/7)	10 mos CGA	Congenital problems, parenchymal hemorrhage, leukomalacia, visual impairment, aspiration precluding oral feeding, or significant social concerns	Schedule for Oral Motor Assessment	20%
Cerro, 2002 [17], Australia	95	29.2 ± 2.1	31 mos CGA (19–43 mos)	Neurological impairment	Informal/Clinical Assessment	73%
Crapnell, 2013 [18], US	80	26.6 ± 1.9	24 mos	Congenital anomalies	Infant – Toddler Social Emotional Assessment – Eating subscale	23%
DeMauro, 2011 [19], US	3 mos CGA - 220 Early PT /401 Late PT 6 mos CGA - 261 Early PT/ 398 Late PT 12 mos CGA - 244 Early PT / 451 Late PT	Early PT: 25–33 6/7 Late PT: 34–36 6/7	3, 6, and 12 mos CGA	Congenital or chromosomal anomalies	Informal/Clinical Assessment	3 mos CGA - Early PT: 33% Late PT: 29% 6 mos CGA - Early PT: 18% Late PT: 20% 12 mos CGA - Early PT: 14% Late PT: 12%
den Boer, 2013 [5], Netherlands	47	30 ± 2	9.6 ± .7 mos CGA	None stated	Informal/Clinical Assessment	47%
Dodrill, 2004 [20], Australia	20	33.9 (32–36)	13.5 mos CGA (11–16.4)	Medical comorbidities	Royal Children’s Hospital Oral Sensitivity Checklist	100%
Enomoto, 2017 [21], Japan	35	23.1–28.6	Term CGA	Abnormal palate at birth	Informal/Clinical Assessment	17.1%
Hawdon, 2000 [22], UK	27	23–37	Term CGA	NICU stay < 5 days and parents with “no fixed abode”	Neonatal Oral Motor Assessment Scale	40.7%
Hoogewerf, 2017 [2], Netherlands	EP: 38 VP: 118 MP: 95	EP: 27 (24–27) VP: 30 (28–31) MP: 34 (32–36)	12–24 mos	NICU care < 4 days, chromosomal anomalies	Montreal Children’s Hospital Feeding Scale – Dutch version	EP: 26.3% VP: 19.5% MP: 15.8%
Johnson, 2016 [4], UK	597	(32–36)	24 mos CGA	Major structural or chromosomal congenital anomalies, cardiovascular malformations and neurosensory impairments	17-item “Validated eating behavior questionnaire”	14.9%
Jonsson, 2013 [23], Sweden	27	31 ± 1.4	< 6 mos	Presence of congenital anomalies or chronic illness not associated with prematurity.	Informal/Clinical Assessment	48%
Kmita, 2011 [24], Poland	Group 1: 22 Group 2: 18	Group 1: 26 (22–29) weeks Group 2: 31 (29–34 weeks)	< 12 mos CGA	Teenage parents or congenital malformations/genetic syndromes	Informal/Clinical Assessment	Group 1: 68.2% Group 2: 55.6%
Mathisen, 2000 [25], Australia	20	27.3 ± 1.65	6–8 mos CGA	IVH, necrotizing enterocolitis, broncho-pulmonary dysplasia, chromosomal abnormality, SGA, receiving supplementary oxygen or tube feeds.	Schedule for Oral Motor Assessment	80%
Nieuwenhuis, 2016 [26], Netherlands	35	30 (26–32)	3–3.9 years CGA	None stated	Montreal Children’s Hospital Feeding Scale – Dutch version	11%
Pridham, 2007 [27], US	41	26.4 ± 1.9 (23–30)	1, 4, 8, and 12 mos corrected GA	Medical conditions that interfere with oral intake of nutrients or small for gestational age at birth	Child Feeding Skills Checklist	1 mos: 28.7% 4 mos: 19.8% 8 mos: 34.5% 12 mos: 41.2%
Sanchez, 2016 [3], Australia	90	27.9 (23.6–29.9)	12 mos CGA	Infants with congenital abnormalities known to affect neurodevelopment	Schedule for Oral Motor Assessment	38%
Sweet, 2003 [28], US	21	24 (22–27)	2 years	Birth weight > 600 g	Informal/Clinical Assessment	62%
Torola, 2012 [29], Finland	19	27 (23–30)	0–5 mos CGA	Congenital or chromosomal anomaly	Neonatal Oral Motor Assessment Scale	100%
Wood, 2003 [30], UK	283	22 1/7–25 6/7	30 mos CGA	No exclusion criteria mentioned	Informal/Clinical Assessment	33%

Note. Additional information about definitions of problematic feeding can be found on Table 2 (Supplementary online content). *EP* Extremely preterm, *CGA* corrected gestational age, *IVH* intraventricular hemorrhage, *mos* months, *MP* moderately preterm, *NICU* neonatal intensive care unit, *PMA* post-menstrual age, *PT* preterm, *SGA* small for gestational age, *VP* very preterm

### Meta-analysis of prevalence

#### Overall prevalence

**Fig. 1:**
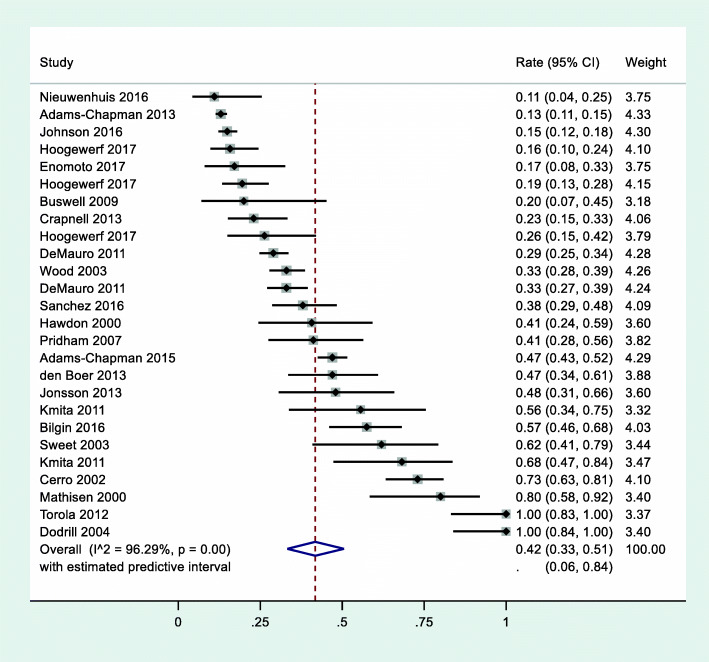
Overall prevalence of problematic feeding in children under 4 years old born preterm across all 22 studies (*N* = 4381)

There were 22 studies that reported prevalence of problematic feeding, which collectively reported on 4381 infants and young children (Fig. 1). Across studies, the overall prevalence of problematic feeding was 42% (95% CI 33–51%; *z* = 14.32; *p* < .01). There was significant (*Q* = 673.94) and substantive (*I*^*2*^ = 96.29%) heterogeneity across studies reporting on the prevalence of problematic feeding. The estimated predictive interval for overall prevalence suggests that future studies, if conducted using similar assessment techniques as used in the included studies, may expect to find a prevalence between 6 and 84%.

#### Prevalence by gestational age at birth

**Fig. 2:**
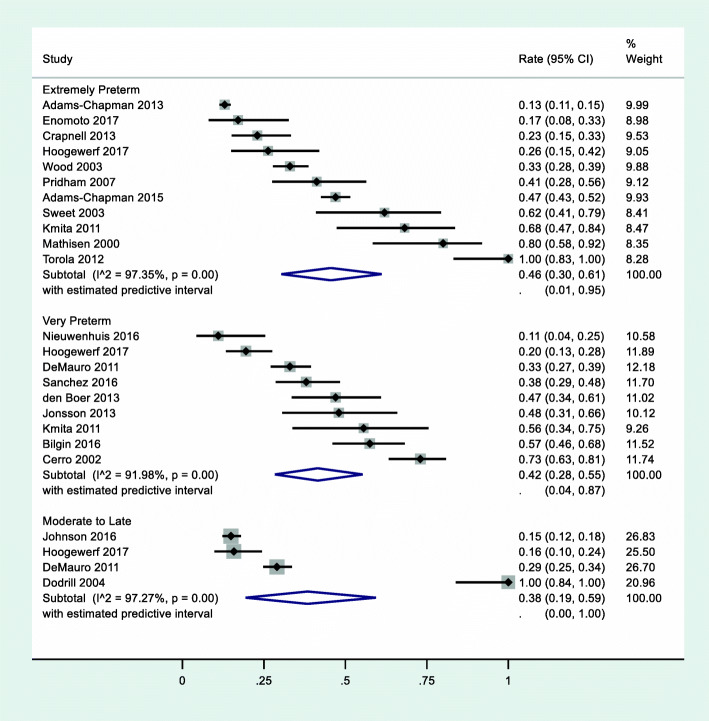
Prevalence of problematic feeding by gestational age at time of birth. Extremely preterm = < 28 weeks gestation at birth (*n* = 2503); very preterm = 28–32 weeks gestation at birth (*n* = 723); moderate to late preterm = 32–37 weeks gestation at birth (*n* = 1113). Note that Buswell et al. [16] and Hawdon et al. [22] were excluded because they reported on infants across multiple gestational age categories at birth

There were 20 studies that reported the prevalence of problematic feeding by gestational age at the time of birth, which collectively reported on 4339 infants (Fig. 2). No statistically significant difference was found in the prevalence of problematic feeding between infants by gestational age at birth (*Q* = .32, *p* = .85); heterogeneity remained high within each group of studies by gestational age at birth (all *I*^*2*^ > 91.98%).

Of the 20 studies included in this analysis, 11 reported on a sample of children born extremely preterm (< 28 weeks gestational age at birth). Among infants born extremely preterm (*n* = 2503), the prevalence of problematic feeding was 46% (95% CI 30–61%, *z* = 8.47; *p* < .01). There was significant (*Q* = 377.79) and substantive (*I*^*2*^ = 97.35%) heterogeneity across studies reporting prevalence of infants < 28 weeks’ gestation at birth.

There were nine studies that reported prevalence on a sample of infants born very preterm (28–32 weeks gestational age at birth) (*n* = 723). Among infants born very preterm, the prevalence of problematic feeding was 42% (95% CI 28–55%, *z* = 9.12; *p* < .01). Within these nine studies, there was significant (*Q* = 99.8) and substantive (*I*^*2*^ = 91.98%) heterogeneity across studies.

There were four studies that reported prevalence of problematic feeding in a sample of moderate to late preterm (gestational age 32–37 weeks) infants (*n* = 1113). The prevalence of problematic feeding in infants born 32–37 weeks’ gestation was 38% (95% CI 19–59%, *z* = 5.72; *p* < .01). Within these four studies, there was significant (*Q* = 109.78) and substantive (*I*^*2*^ = 97.27%) heterogeneity across studies.

#### Prevalence by child age at time of study

**Fig. 3:**
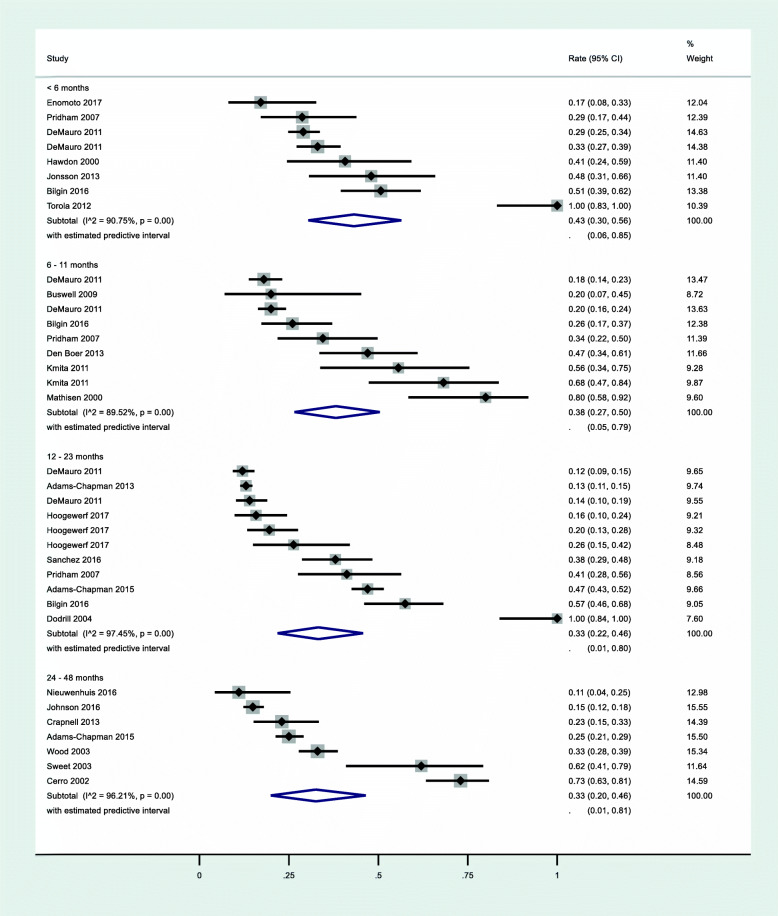
Prevalence of problematic feeding by child age at time of study

Within the 22 studies reporting on prevalence of problematic feeding in young children, seven studies reported prevalence in children aged 0–5 months at the time of study, seven studies reported on children aged 6–11 months at the time of study, 8 studies reported on children aged 12–23 months, and 7 studies reported on children 24–48 months. Several studies were longitudinal in nature and reported on problematic feeding of the same children at multiple ages, thus these samples were not entirely independent. There was no statistically significant difference found in prevalence of problematic feeding between young children of different ages at the time of study (*Q* = 1.73, *p* = .63) (Fig. 3); heterogeneity remained high within each group of studies by age at time of assessment (all *I*^*2*^ > 89.52%).

Problematic feeding occurred with a prevalence rate of 43% (95% CI 30–56%, *z* = 9.69; *p* < .01) in infants aged 0–5 months (*n* = 843). Within the seven studies reporting on eight different groups of infants in this age group, there was significant (*Q* = 75.66) and substantive (*I*^*2*^ = 90.75%) heterogeneity across studies.

In infants aged 6–11 months (*n* = 895) born prematurely, the prevalence of problematic feeding was 38% (95% CI 27–50%, *z* = 9.49; *p* < .01). Within the seven studies reporting on nine different groups of infants in this age group, there was significant (*Q* = 76.31) and substantive (*I*^*2*^ = 89.52%) heterogeneity across studies.

The prevalence of problematic feeding among toddlers aged 12–23 months (*n* = 3114) was 33% (95% CI 22–46%, *z* = 8.66; *p* < .01). Within the eight studies reporting on 11 different groups of toddlers in this age group, there was significant (*Q* = 392.73) and substantive (*I*^*2*^ = 97.45%) heterogeneity across studies.

Finally, among children 24–48 months old (*n* = 1578), the prevalence of problematic feeding was 33% (95% CI 20–46%, *z* = 7.61; *p* < .01). Among the seven studies reporting on seven groups of children in this age group, there was significant (*Q* = 158.42) and substantive (*I*^*2*^ = 96.21%) heterogeneity across studies.

### Assessment of Bias of included studies

#### Quality of feeding assessment

Of the 22 studies included in this meta-analysis, only five utilized a formal assessment of feeding with evidence of strong psychometric properties [2, 3, 16, 25, 26]. Three studies used the Schedule for Oral Motor Assessment (SOMA) [3, 16, 25], an assessment conducted from a video recording of a structured feeding session. In the context of the SOMA, children are offered a series of food challenges with varying textures and degrees of difficulty to evaluate oral-motor function. The SOMA has evidence of strong psychometric properties, including acceptable test-retest reliability [32, 33], predictive validity [34], criterion validity [34], and intra-rater and inter-rater reliability [33, 35, 36].

Two studies used the Montreal Children’s Hospital Feeding Scale (MCHFS) - Dutch version [2, 26]. The MCHFS is a 14-item parent-report tool that assesses multiple constructs, including oral motor, oral sensory, appetite, maternal concerns about feeding, mealtime behaviors, maternal strategies, and family reactions to the child’s feeding [37]. Of the 14 items on the MCHFS, only nine items relate to the child’s behavior or skill during feeding. The original bilingual version (in English and French) has evidence of known-groups validity and test-retest reliability [37], as well as internal consistency reliability (Cronbach’s α = .9) and construct validity with related measures [38]. The Dutch version of the MCHFS, called the Screeninglijst Eetgedrag Peuters, also has evidence of acceptable internal consistency reliability (Cronbach’s α = .75–.84) [39] and concurrent validity with clinical assessment in both children born premature [40] and with Down Syndrome [41].

Five of the 22 studies included in the meta-analysis assessed feeding using a formalized assessment with inconsistent evidence of psychometric properties or limited psychometric testing. Two of these five studies [22, 29] used the Neonatal Oral Motor Assessment Scale (NOMAS) [42], which is a 28-item clinician-report assessment of jaw and tongue movement and function. The psychometric properties of the NOMAS have been tested in multiple research studies, but with inconsistent results with regards to inter-rater and test-retest reliability [43–47], as well as poor evidence of construct validity [46, 47]. Psychometric properties of the NOMAS improved after a change was made to the scoring system in 2016 [48], but both of the studies included in this meta-analysis were conducted prior to this change.

Crapnell and colleagues [18] used the 9-item Infant-Toddler Social Emotional Assessment (ITSEA) – Eating subscale [49], which assessed gagging and choking, eating refusal, spitting of food, picky eating, and holding food in the cheek. The ITSEA – Eating subscale has reported acceptable internal consistency reliability (Cronbach’s α = .78–.82) [18, 49]. The full ITSEA scale has reported acceptable test-retest reliability, interrater reliability, and evidence of criterion validity [49], however these data have not been reported specifically for the ITSEA – Eating subscale.

Johnson and colleagues [4] used a 17-item eating behavior questionnaire [50] that assessed four domains of eating difficulties, including refusal/picky eating, oral motor problems, oral hypersensitivity, and eating behavior problems. This questionnaire had documented acceptable internal consistency reliability for the full measure (Cronbach’s α = .83–.88) [4, 50], as well as for three of the four subscales (Cronbach’s α = .79–.9) [50]. The 4-item subscale on eating behavior problems had a reported internal consistency reliability (Cronbach’s α = .55) [50] that was below the generally accepted threshold of .7 [51]. No other psychometric testing of validity or reliability has been published. Finally, Bilgin and Wolke used a 7-item scale they created to assess “faddy eating” (i.e., picky eating) and food refusal [15] and included items related to eating too little, having a poor appetite, eating slowly, being sensitive to textures, and picky eating. No information was provided on the development of these items, but internal consistency reliability was reported as acceptable (Cronbach’s α = .74–.81) [15].

Two of the 22 studies included in the meta-analysis used formalized feeding assessments but with no published psychometric properties. Pridham and colleagues [27] used the Child Feeding Skills Checklist, which is an observational tool. For the purposes of this meta-analysis, we utilized information reported on observed oral-motor skills. Additionally, Dodrill and colleagues [20] reported on oral sensitivity using the Royal Children’s Hospital Oral Sensitivity Checklist. While this is a more formalized assessment of facial defensiveness and sensitivity to oral stimulation, there are no published psychometrics on this measure.

Of the 22 included studies, 10 used an informal or clinical assessment of feeding with no psychometric testing. The ways in which these 10 studies defined problematic feeding varied widely. The specific definitions of problematic feeding used in these 10 studies are provided on Table 2. For example, Adams-Chapman and colleagues [14] defined dysfunctional feeding as a physician order not to ingest feedings by mouth, any need for gastrostomy or tube feedings, gagging, choking, or coughing with oral feeding, documented history of aspiration, excessive drooling during feeding, or difficulty swallowing. Enomoto [21], on the other hand, considered feeding to be a problem if the infant required a milk-thickening agent, but they did not describe the process for determining the need for a milk-thickening agent. Other informal assessments included questions about appetite, oral-motor dysfunction, avoidant feeding behaviors, choking, gagging, excessive spit-up, and difficulties during feeding observed by the provider.

**Table 2:** Problematic Feeding Definitions and Prevalence Calculations

First Author, Year of Publication	Feeding Assessment	Definition of Problematic Feeding	Prevalence Calculation
Adams-Chapman, 2013 [14]	Informal/Clinical Assessment	Dysfunctional feeding defined as: 1) medical order not to ingest feedings by mouth, 2) need for gastrostomy or tube feedings, 3) gags/chokes or coughs with feeds, 4) documented aspiration, 5) excessive drooling during feeds, or 6) difficulty swallowing.	13% of the sample were reported to have dysfunctional feeding per the definition at left.
Adams-Chapman, 2015 [13]	Informal/Clinical Assessment	Abnormal feeding defined as: unable to tolerate foods by mouth, requiring tube feeds for > 50% of nutritional intake, or choking, gagging, coughing, or gasping with solids. Drooling continuously or having documented history of dysphagia or aspiration were also considered abnormal.	47% had abnormal feeding at 18 months. 25% had abnormal feeding at 30 months.
Bilgin, 2016 [15]	Informal/Clinical Assessment and Faddy Eating/Food Refusal Scale	*Problems in oral motor functioning* were measured with the following items: (1) stopping after a few sucks, (2) excessive dribbling/difficulty swallowing, and (3) gagging/choking during the feed. Participants who endorsed 2 or 3 items were determined to have an oral-motor function problem. *Faddy eating/food refusal*: At term, 3, and 6 months, endorsement of the following item was deemed faddy eating/food refusal: fighting against the bottle/breast. At 18 months, a faddy eating/food refusal scale included the following variables: Eats too little, leaves most of the food offered, poor appetite, picky eater, slow eater, refuses to eat lumpy food, or refuses to eat pureed food. Participants who endorsed 5 or more problems were determined to have faddy eating/food refusal.	Feeding problems were defined as having an oral-motor function problem and/or faddy eating/food refusal (per definitions at left). These authors found the following prevalence of feeding problems at each age: Term – 50.7%; 3 months – 20.5%; 6 months – 26%; and 18 months – 57.5%.
Buswell, 2009 [16]	Schedule for Oral Motor Assessment (SOMA)	Infants who scored on or above the threshold on the SOMA were determined to have oral motor dysfunction.	20% of the sample were found to have oral-motor dysfunction.
Cerro, 2002 [17]	Informal/Clinical Assessment	Parents’ perceptions of their child’s eating behavior were explored using an unpublished 48-item questionnaire developed in consultation with various experts and consideration of current literature. The questionnaire involved closed questions, Likert scales and specified lists.	73% of the sample was reported to have at least one feeding problem.
Crapnell, 2013 [18]	Infant – Toddler Social Emotional Assessment – Eating subscale	Children were determined to have a feeding problem if their score exceeded the ≥10th percentile of the normative sample.	23% of the sample met criteria for a feeding problem.
DeMauro, 2011 [19]	Informal/Clinical Assessment	A feeding behavior questionnaire was used that included 4 questions about oromotor dysfunction and 7 questions about avoidant feeding behavior. Participant scores were categorized as normal/low if no items were endorsed, borderline/medium if 1 item was endorsed, and high if 2 or more of the items were endorsed.	At 3 months, 33% of early preterm infants and 29% of late preterm infants had medium or high avoidant behavior. At 6 months, 18% of early preterm infants and 20% of late preterm infants had medium or high avoidant behavior. At 12 months, 14% of early preterm infants and 12% of late preterm infants had medium or high avoidant behavior.
den Boer, 2013 [5]	Informal/Clinical Assessment	Eating and drinking observed by a speech and language therapist. The feeding was then rated for: choking while drinking, choking while eating, and gagging.	40% of the sample was found to have choking while drinking. 46% of the sample was found to have choking while eating. 55% of the sample was found to have gagging during a meal. For the meta-analysis, we used an average of these problems for a prevalence of 47% of the preterm sample having feeding-related problem.
Dodrill, 2004 [20]	Royal Children’s Hospital Oral Sensitivity Checklist	A subset of questions on the Royal Children’s Hospital Oral Sensitivity Checklist evaluates the child’s response to stimulation of the oral region. Abnormal sensitivity was defined as any behavior suggesting abnormal sensitivity, included behaviors such as head turning, gagging, and vomiting with oral stimulation.	100% of the sample displayed behaviors suggestive of abnormal oral sensitivity.
Enomoto, 2017 [21]	Informal/Clinical Assessment	A feeding problem was defined as oral feeding difficulty requiring a milk-thickening agent.	17.1% of the sample required a milk-thickening agent.
Hawdon, 2000 [22]	Neonatal Oral Motor Assessment Scale (NOMAS)	Feeding pattern was categorized according to the NOMAS as normal, disorganized, or dysfunctional.	Of the 27 preterm infants in the sample, 11(i.e., 40.7%) were found to have either disorganized or dysfunctional feeding.
Hoogewerf, 2017 [2]	Montreal Children’s Hospital Feeding Scale (MCHFS)– Dutch version	Children were categorized as having a feeding problem if their score on the MCHFS was > 1 standard deviation from the mean score of the reference population.	Of the children born extremely preterm, 26.3% of the sample were found to have a feeding problem. 19.5% of the children born very preterm were found to have a feeding problem, while 15.8% of children born moderately preterm were found to have a feeding problem.
Johnson, 2016 [4]	17-item “Validated eating behavior questionnaire”	Total eating difficulties score was calculated for the 17-item questionnaire. A score > 90th percentile of the term, control group was considered significant eating difficulties.	14.9% of the late- and moderately-preterm group were found to have significant eating difficulties.
Jonsson, 2013 [23]	Informal/Clinical Assessment	Questionnaire developed by authors - Symptoms measured included vomiting, eating reluctance, poor weight gain, long feeding time, or other symptoms.	48% of the preterm group were reported to have had some form of difficulty with feeding at the time of being discharged from the hospital.
Kmita, 2011 [24]	Informal/Clinical Assessment	Parental descriptions of feeding behavior were explored through exploratory analysis of semi structured interviews and daily diaries.	Parents reported no problems with feeding in 31.8% of group 1 and 44.4% of group 2. We then calculated that 68.2% of group 1 and 55.6% of group 2 had some problematic feeding behavior.
Mathisen, 2000 [25]	Schedule for Oral Motor Assessment (SOMA)	Scores on or above the threshold of the SOMA met criteria for oral motor dysfunction.	80% of the extremely low birth weight infants were reported to have feeding problems.
Nieuwenhuis, 2016 [26]	Montreal Children’s Hospital Feeding Scale (MCHFS) – Dutch version	Scores on the MCHFS – Dutch version >84th percentile met criteria for a feeding problem.	11% of the preterm sample was found to have a score on the MCHFS – Dutch version that met criteria for a feeding problem.
Pridham, 2007 [27]	Child Feeding Skills Checklist	Preterm-born children were observed using the Child Feeding Skills Checklist to assess feeding skills that would be expected at 1, 4, 8, and 12 months corrected gestational age.	Pridham and colleagues reported the percent of the sample that was able to perform the oral-motor skills expected at each time of measure. From this, we calculated the average percent of the sample at each time of measure that was not able to perform the expected oral-motor skill, and therefore determined to have some degree or problematic feeding. At 1 month, an average of 28.7% were not able to perform the expected oral-motor skills. At 4 months, an average of 19.8% were not able to perform expected skills. At 8 months, an average of 34.5% were not able to perform expected skills. At 12 months, an average of 41.2% were not able to perform expected skills.
Sanchez, 2016 [3]	Schedule for Oral Motor Assessment (SOMA)	Oral motor feeding impairment was defined as failing ≥1 SOMA challenge.	38% of the sample of preterm children failed ≥1 SOMA challenge.
Sweet, 2003 [28]	Informal/Clinical Assessment	“Feeding problems” observed during neonatal follow-up clinic visit.	13 of the 21 infants (i.e., 62%) who returned for the 2 year follow-up were found to have feeding problems.
Torola, 2012 [29]	Neonatal Oral Motor Assessment Scale (NOMAS)	The NOMAS categorizes the sucking pattern into normal, disorganized or dysfunctional.	None of the preterm infants had a normal sucking pattern. 84.2% had disorganized sucking while 15.8% had a dysfunctional sucking pattern.
Wood, 2003 [30]	Informal/Clinical Assessment	Feeding history obtained using a semi-structured interview.	Parents of 33% of children reported feeding difficulties at 30 months corrected gestational age.

*MCHFS* Montreal Children’s Hospital Feeding Scale (MCHFS), *NOMAS* Neonatal Oral Motor Assessment Scale, *SOMA* Schedule for Oral Motor Assessment

### Sampling Bias of included studies

Exclusion criteria for each study are presented on Table 1. The most common exclusion criteria from the 22 included studies were congenital anomalies/malformations (41%) and congenital syndromes/genetic disorders/chromosomal anomalies (36%). Five studies (23%) excluded infants with neurological abnormalities, including acquired conditions related to prematurity, and four studies (18%) did not define or report their exclusion criteria. Less common reasons from exclusion from the sample were social concerns (14%), congenital infections (9%), short stays in the neonatal intensive care unit (NICU; 9%) and small for gestational age at birth (9%). Rare reasons were exclusion included visual impairment, aspiration precluding oral feeding, medical comorbidities, abnormal palate, chronic illness not associated with prematurity, necrotizing enterocolitis, bronchopulmonary dysplasia, requiring supplemental oxygen or tube feedings, and medical conditions that interfere with oral intake of nutrients.

## Discussion

This meta-analysis of currently available data found that problematic feeding was highly prevalent (42%) in children under 4 years of age who were born prematurely (< 37 weeks’ gestation). The main limitation of the data included in these analyses was that few of the studies used formalized assessments of feeding with evidence of adequate psychometric properties. Our finding of significant and substantive heterogeneity across studies likely reflects this issue of poor measurement of the problem, as well as variation in the samples studied and true variation of problematic feeding in infants with varying degrees of medical complexity. Across studies, the definition of problematic feeding varied widely and, in many cases, only captured those with feeding difficulties on the more severe end of the spectrum.

Even in the studies that did use a psychometrically-sound formalized assessment of feeding, there were limitations of the assessments used. The SOMA, which was used by three studies and had the most evidence of psychometric integrity, is a measure focused specifically on oral-motor function. While oral-motor function is a critical component of feeding, comprehensive assessment of feeding also includes evaluation of physiologic stability, behavioral responses to feeding, swallowing, gastrointestinal tract function, and ability to regulate satiety and hunger. Evaluation of problematic feeding by SOMA alone is likely to underestimate the true prevalence of feeding problems.

The MCHFS, utilized by two of the studies included in this meta-analysis, is a more inclusive assessment than the SOMA and includes evaluation of oral-motor function, oral sensory function, appetite, and mealtime behaviors. However, with only nine items directly related to the child’s eating, it is not a comprehensive assessment. The MCHFS also mixes in constructs related to maternal concerns about feeding, maternal strategies, and family reactions to the child’s feeding. While these are important factors in an overall assessment of the family and can be highly related to problematic feeding in the child, these are complex constructs that should be evaluated separately from an assessment of the child’s ability and willingness to eat. When these constructs are mixed within the same assessment, it is unclear whether abnormal score reflects a problem related to the child’s ability or willingness to eat or whether the score reflects difficulty in family functioning, family stress, maternal coping, or education about feeding.

In addition to the poor measurement of problematic feeding, many of the studies included in this meta-analysis excluded children who were at highest risk for developing problematic feeding. Many studies excluded infants with congenital infections and anomalies, as well as those with neurologic impairment, a common comorbidity associated with premature birth. For example, Mathisen and colleagues [25] reported 80% of their sample of very preterm infants had problematic feeding using the SOMA, even when excluding those with common complications of prematurity that are likely to increase risk of feeding difficulties, including intraventricular hemorrhage, necrotizing enterocolitis, bronchopulmonary dysplasia, and need for supplementary oxygen or tube feedings. Given that those premature infants with highest risk for problematic feeding were excluded from many studies, it may be that the overall prevalence of 42% identified from this meta-analysis is an underestimate of the true prevalence of problematic feeding in all children born premature. This also means that problematic feeding is a complication of premature birth in approximately 42% of children who may otherwise be considered lower risk because they do not have other major comorbidities.

When we explored the prevalence of problematic feeding by gestational age at birth, our analyses found no statistically significant difference. This finding is consistent with that of Hoogewerf and colleagues [2], included in this meta-analysis, who used the MCHFS and found no difference in prevalence of problematic feeding by gestational age. However, this finding is not consistent with other literature. In a study of 256 children born premature, Park and colleagues [6] found that children born very preterm had significantly more feeding problems than children born moderate to late preterm. This inconsistency in the literature is likely a reflection of the measurement of feeding problems. Park and colleagues [6] used the Pediatric Eating Assessment Tool, which is a comprehensive measure of feeding with strong evidence of psychometric properties that only measures symptoms of problematic feeding and does not mix constructs of feeding strategies or family concerns [10, 52, 53]. Of note, the study by Park and colleagues was not included in this meta-analysis because the prevalence of problematic feeding within the sample was not reported.

Our analyses also found no difference in prevalence of problematic feeding by the child’s age at the time of study. This finding was also not consistent with the findings of Park and colleagues [6], who found that preterm-born children aged 6–15 months had significantly more feeding problems than those aged 15 months to 2.5 years. This inconsistency is likely a result of better measurement in the Park [6] study and/or differences in the categorization of child ages.

### Limitations

As discussed, the main limitations at the study and outcome level were related to few studies using psychometrically-sound assessments of feeding and exclusion of children with highest risk for problematic feeding. At the review level, the data used for this meta-analysis was limited to studies found by searching PubMed, CINAHL, and PsycInfo and available in English language through the global inter-library loan network available to the first author. It is possible that additional research studies reporting on prevalence of problematic feeding in children born prematurely are available outside of these databases, in other languages, or through other networks.

## Conclusions

Problematic feeding occurs in approximately 42% of children under 4 years of age who were born prematurely (< 37 weeks’ gestation). To date, the study of problematic feeding in children has been limited by a lack of definition of the problem [12] and lack of valid and reliable measures. In 2019, Goday and colleagues proposed a consensus definition of Pediatric Feeding Disorder - impaired oral intake that is not age-appropriate, and is associated with medical, nutritional, feeding skill, and/or psychosocial dysfunction [12]. This improved definition of the problem, along with newly-developed, psychometrically-sound measures of feeding [7–11, 52–59] can be used to improve upon the research and care of problematic feeding in children born preterm and with other medical conditions. A large epidemiological study using a comprehensive and psychometrically-sound assessment of feeding is needed to determine the true prevalence of problematic feeding in children born preterm.

## Supplementary Information

**Additional file 1: Figure 1.** Preferred Reporting Items for Systematic Reviews and Meta-Analyses (PRISMA) diagram of study source identification, screening, inclusion, and exclusion (with reasons).

**Additional file 2.**

## Data Availability

The datasets used and/or analysed during the current study are available from the corresponding author on reasonable request.

## Abbreviations

CGA: Corrected gestational age
EP: Extremely preterm
ITSEA: Infant-Toddler Social Emotional Assessment
IVH: Intraventricular hemorrhage
MCHFS: Montreal Children’s Hospital Feeding Scale
Mos: Months
MP: Moderately preterm
NICU: Neonatal intensive care unit
NOMAS: Neonatal Oral Motor Assessment Scale
PMA: Post-menstrual age
PRISMA: Preferred Reporting Items for Systematic Reviews and Meta-Analyses
PT: Preterm
SGA: Small for gestational age
SOMA: Schedule for Oral Motor Assessment
VP: Very preterm
