# Supplementary figures and images for: Prevalence of problematic feeding in young children born prematurely: a meta-analysis
Source: BMC Pediatr. 2021 Mar 6;21:110. doi: 10.1186/s12887-021-02574-7 (PMC7936467; doi:10.1186/s12887-021-02574-7)

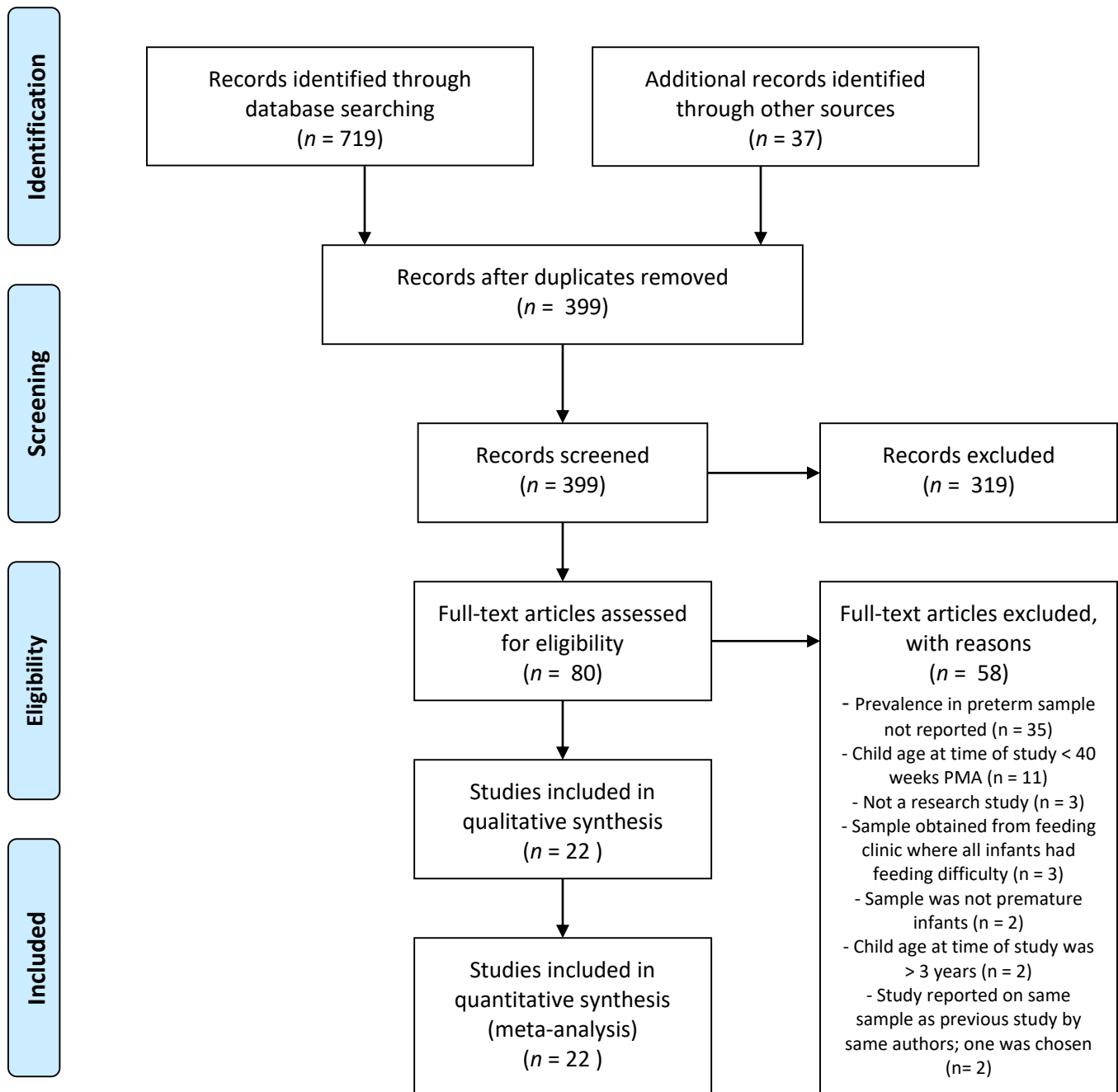

Supplement: Supplementary file 1 — Additional file 1: Figure 1. Preferred Reporting Items for Systematic Reviews and Meta-Analyses (PRISMA) diagram of study source identification, screening, inclusion, and exclusion (with reasons). [file 12887_2021_2574_MOESM1_ESM.pdf]
